# Mitotic Regulation by NEK Kinase Networks

**DOI:** 10.3389/fcell.2017.00102

**Published:** 2017-12-01

**Authors:** Andrew M. Fry, Richard Bayliss, Joan Roig

**Affiliations:** ^1^Department of Molecular and Cell Biology, University of Leicester, Leicester, United Kingdom; ^2^School of Molecular and Cellular Biology, University of Leeds, Leeds, United Kingdom; ^3^Institut de Biologia Molecular de Barcelona (IBMB-CSIC), Barcelona, Spain

**Keywords:** protein kinase, mitosis, microtubule, centrosome, cilia

## Abstract

Genetic studies in yeast and *Drosophila* led to identification of cyclin-dependent kinases (CDKs), Polo-like kinases (PLKs) and Aurora kinases as essential regulators of mitosis. These enzymes have since been found in the majority of eukaryotes and their cell cycle-related functions characterized in great detail. However, genetic studies in another fungal species, *Aspergillus nidulans*, identified a distinct family of protein kinases, the NEKs, that are also widely conserved and have key roles in the cell cycle, but which remain less well studied. Nevertheless, it is now clear that multiple NEK family members act in networks to regulate specific events of mitosis, including centrosome separation, spindle assembly and cytokinesis. Here, we describe our current understanding of how the NEK kinases contribute to these processes, particularly through targeted phosphorylation of proteins associated with the microtubule cytoskeleton. We also present the latest findings on molecular events that control the activation state of the NEKs and how these are revealing novel modes of enzymatic regulation relevant not only to other kinases but also to pathological mechanisms of disease.

## NEK kinases: mitosis, cilia, and more

The founding member of the NIMA family was isolated in the 1980s in a loss-of-function genetic screen for regulators of the cell cycle in the filamentous fungus *Aspergillus nidulans*. Two classes of mutant were identified: those that became blocked in mitosis, so-called “*bim*” mutants, and those that were blocked in interphase and were therefore never in mitosis, so called “*nim*” mutants (Oakley and Morris, 1983). The first of the latter class of mutants to be studied, *nimA*, was found to encode a protein kinase (Osmani et al., 1988). Further work showed that wild-type NIMA regulates several key events of mitosis including chromosome condensation, nuclear envelope breakdown and spindle organization (O'Connell et al., 2003). Importantly, NIMA-related kinases, or NEKs (also referred to in some species as NRKs), are conserved across most eukaryotes. NEKs share a protein kinase domain, usually located at the N-terminus of their sequence, that define them as members of the family. Most also possess a non-catalytic domain, but these are largely unrelated in sequence, length and organization, and thereby able to confer specific mechanisms of upstream regulation and downstream substrate selection. Furthermore, while some NEKs in higher eukaryotes act like *Aspergillus* NIMA to control mitotic progression, other members of this family have acquired functions elsewhere in the cell cycle (Moniz et al., 2011; Fry et al., 2012).

Strikingly, the number of NEKs encoded within the genome varies widely from organism to organism. Intriguingly, this number correlates with complexity of the molecular structures that contribute to formation of cilia and flagella, which typically involves a basal body or centrosome. This has led to the suggestion that the NEK family evolved in parallel with the ciliary apparatus such that organisms that require a more complex regulation of this structure have expanded the number of NEKs diversifying their functions according to their specific needs (Quarmby and Mahjoub, 2005; Parker et al., 2007). Indeed, non-ciliated yeast and molds (e.g., *Saccharomyces, Schizosaccharomyces*, and *Aspergillus*) encode a single NEK kinase, while the amoebozoa *Dictyostelium* that also lacks classical centrioles and cilia has only two. *Trypanosoma* and *Chlamydomonas*, with respectively one and two flagella, have ten NEKs and the multiciliated *Tetrahymena* almost forty. *Plasmodium* is phylogenetically related to *Tetrahymena* but expresses only four NEKs. However, three of these are restricted to gametocytes and this is consistent with the male gamete being the only cell with flagella.

Remarkably, *Giardia*, with a complex array of four pairs of specialized flagella, has almost 200 NEKs that represent more than two thirds of the kinases encoded in its genome (and almost 4% of its proteome). The surprising expansion of the NEK family in *Giardia* is yet to be explained. The high number of NEKs may be related to the needs of controlling four pairs of different cilia, and their corresponding basal bodies, that are inherited in a specific pattern during cell division. However, this is unlikely to be the sole reason as *Giardia* NEKs are highly diverse and the majority look by sequence to be enzymatically-inactive pseudokinases. On the other hand, most of these NEKs are expressed and localize in specific manners suggesting that they retain a function. In addition, NEKs appear to be undergoing rapid evolution in these organisms based on observable changes between strains, implying that NEKs may have a role in establishment of strain-specific differences (Manning et al., 2011).

In vertebrates, most cells assemble a single immotile primary cilium that is involved in sensory signaling, but a few specialized cell types exist, such as respiratory epithelia, ependymal cells and sperm, that have one or more cilia with mechanical functions (Ishikawa and Marshall, 2011). Consistent with the model for co-evolution with the microtubule organization apparatus, vertebrates have an intermediate number of NEKs with humans possessing eleven, named Nek1 to Nek11 (Moniz et al., 2011; Fry et al., 2012). Nek2, Nek5, Nek6, Nek7, and Nek9 have different functions related to control of the centrosome cycle (see below), while Nek1 and Nek8 are involved in regulation of cilia physiology (Upadhya et al., 2000; Liu et al., 2002; Otto et al., 2008; Shalom et al., 2008; Zalli et al., 2012). Importantly, a number of the human NEKs have been found to be mutated in ciliopathies, inherited disorders that result from defective organization and/or function of the primary cilium (Hildebrandt et al., 2011).

Although plant cells generally lack centrioles or cilia, they do have around six NEKs; but all belong to a single group related to human Nek6 and Nek7. *Drosophila*, in which only sperm cells and sensory neurons have cilia, has only two NEKs (Nek2 and Niki, in the Nek8/Nek9 group), while *C. elegans*, with amoeboid sperm and where only sensory neurons are ciliated, has four (Nekl-1-4, homologous to mammalian Nek9, Nek8, Nek6/Nek7, and Nek10, respectively). Interestingly, planarians have lost centrosomes and only assemble centrioles and cilia in terminally differentiated cells, yet they retain several NEKs, including Nek6/Nek7 and Nek8/Nek9 homologs (Azimzadeh et al., 2012). However, they have lost Nek2, as well as proteins involved in centrosome duplication, suggesting that the main function of this kinase—the closest vertebrate homolog to *Aspergillus* NIMA—relates to centrosome regulation.

Consistent with their broad function at centrioles and cilia, NEKs are frequently localized to sites of microtubule organization. This is the case in the unicellular *Chlamydomonas, Trypanosoma, Tetrahymena*, and *Giardia* (Mahjoub et al., 2004; Pradel et al., 2006; Wloga et al., 2006; Manning et al., 2011), as well as metazoa. However, in parallel to the proposed ancestral role at the ciliary apparatus, different family members have acquired novel, and sometimes unrelated, functions. Thus, in *Aspergillus*, besides being central to the regulation of the spindle pole body (SPB) and mitotic spindle, NIMA controls chromatin condensation (De Souza et al., 2000), while a growing number of NEKs have been implicated in the DNA damage response. This is the case for yeast Kin3 and several mammalian NEKs (Noguchi et al., 2002; Lee et al., 2008; Moura et al., 2010; Moniz and Stambolic, 2011; Nguyen et al., 2012; Jackson, 2013; Liu et al., 2013; Tan et al., 2017). In addition some of the mammalian NEKs have acquired tissue-specific functions, such as Nek7 in macrophages or Nek3 and Nek7 in neurons (Chang et al., 2009; He et al., 2016; Schmid-Burgk et al., 2016; Shi et al., 2016). Other unexpected functions no doubt await discovery.

## NEK kinases in centrosome disjunction

**Figure 1:**
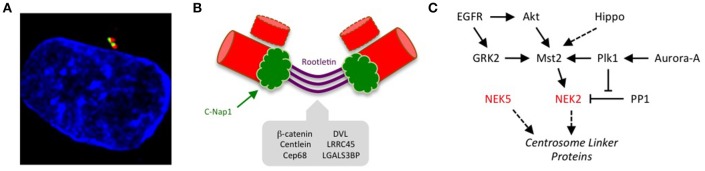
Kinase-mediated pathways regulating centrosome disjunction. **(A)** An immunofluorescence micrograph of a human U2OS osteosarcoma cell stained with acetylated-tubulin antibodies to detect centrioles (red), C-Nap1 antibodies to detect the centrosome linker (green) and Hoechst 33258 to detect DNA (blue). This illustrates how the centrosome appears as a paired structure that sits in the cytoplasm close to the nucleus in most interphase cells. **(B)** A schematic cartoon showing how the centrosome linker is thought to extend between the proximal ends of the two parental centrioles throughout interphase, both before (G1) and during (S/G2) the process of centriole duplication. The best-characterized linker proteins are C-Nap1, which associates with proximal ends of the centrioles, and rootletin, which forms connecting filaments between the centrioles. Additional proteins known to localize to the linker are indicated. **(C)** The centrosome linker undergoes disassembly at the onset of mitosis as a result of activation of Nek2 and phosphorylation of linker proteins. The activation of Nek2 is tightly controlled by a network of other kinases and phosphatases as indicated.

The first clue that NEKs may contribute to microtubule organization came from discovery that human Nek2 localizes to the centrosome (Fry et al., 1998b). Nek2 is a cell cycle-regulated kinase with maximal expression and activity in S and G2 (Fry et al., 1995). Overexpression of wild-type Nek2 induced the unscheduled separation of centrosomes during interphase, while overexpression of a catalytically-inactive mutant interfered with centrosome separation upon entry into mitosis (Fry et al., 1998b; Faragher and Fry, 2003). At the time these observations were made, the mechanisms of centrosome positioning within cells was rather poorly understood. The central location close to the nucleus was initially thought to result from combined centripetal forces of the microtubule and actin networks, as well as protein-based tethers that link the centrosome to the nucleus (Burakov et al., 2003). However, the purification of centrosomes from cultured cells using sucrose gradient fractionation led to the isolation of paired structures providing unequivocal evidence for the presence of a physical connection or “linker” between the two centrosomes (Bornens et al., 1987). Hence, the data on Nek2 fit the hypothesis that its kinase activity causes disassembly of the centrosome linker in a process that has now been termed centrosome disjunction (Agircan et al., 2014; Figure 1).

Support for this hypothesis came from discovery of the first centrosome linker component, C-Nap1 (also called CEP250), using a yeast two-hybrid screen to isolate Nek2 binding partners (Fry et al., 1998a). C-Nap1 is an excellent substrate of Nek2 with many sites of phosphorylation (Mardin et al., 2010; Hardy et al., 2014; Cervenka et al., 2016). Furthermore, C-Nap1 concentrates at centrosomes in interphase cells but is absent from spindle poles in mitosis; it is also substantially reduced at the split centrosomes seen in interphase cells overexpressing Nek2. These properties meet the criteria for a centrosome linker component regulated by Nek2 at the G2/M transition. Indeed, subsequent experiments have revealed that cells lacking Nek2 fail to remove C-Nap1 from centrosomes upon mitotic entry (Fletcher et al., 2004; Mardin et al., 2010), while loss of C-Nap1 by antibody microinjection, RNAi-mediated depletion, gene-editing or disease-associated truncating mutations all lead to unscheduled separation of centrosomes in interphase (Mayor et al., 2000; Bahe et al., 2005; Floriot et al., 2015; Panic et al., 2015; Flanagan et al., 2017).

C-Nap1 is a relatively large (~280 kDa) protein that is almost exclusively composed of predicted coiled-coil motifs. It specifically localizes to the proximal ends of centrioles, where the centrosome linker is assumed to attach. Here, it can bind Cep135, a centriole cartwheel component, with interaction occurring between the C-terminal regions of each protein (Kim et al., 2008). Phosphorylation of C-Nap1 by Nek2 inhibits interaction with Cep135, most likely through electrostatic repulsion (Hardy et al., 2014). While C-Nap1 is displaced from centrosomes upon mitotic entry, Cep135 remains present throughout the cell cycle, supporting the hypothesis that it is loss of the physical connection between these two proteins that triggers centrosome disjunction. However, the molecular nature of the interaction between C-Nap1 and Cep135, and whether this is sufficient to explain how the linker attaches to centrioles, remain important areas for future research.

C-Nap1 is by no means the only component of the centrosome linker. Indeed, immuno-electron microscopy experiments suggest that it is restricted to centriole proximal ends, and not present along the length of the linker (Fry et al., 1998a; Mayor et al., 2002). The core of the linker seems rather to be generated by rootletin, a protein that also forms the major component of the ciliary rootlet (Yang et al., 2002). Ciliary rootlets are well-ordered fibers that extend from the basal body, an equivalent structure to the centrosome, into the inner cell body of photoreceptors. However, rootletin is also present on interphase centrosomes in proliferating cells where it forms elongated fibers attached to the centrosome (Bahe et al., 2005; Yang et al., 2006). Like C-Nap1, rootletin is displaced from centrosomes upon mitotic entry, consistent with the expected behavior of a linker protein. In fact, rootletin is highly related in sequence to C-Nap1 and can oligomerise both with itself and with C-Nap1 through multiple regions (Yang et al., 2006). This has led to the model that C-Nap1 forms the ends of the linker, while rootletin assembles into entangling filaments that compose the core of the linker. FRAP analysis reveals that the linker is a relatively stable structure and its disassembly upon entry into mitosis is not associated with degradation of either C-Nap1 or rootletin (Mayor et al., 2002). Indeed, like C-Nap1, rootletin is phosphorylated by Nek2, and together these linker proteins provide the bulk of recruitment sites for Nek2 at the centrosome (Bahe et al., 2005; Hardy et al., 2014). Hence, the current view is that phosphorylation by Nek2 disturbs not only interaction of C-Nap1 and Cep135, but also attachment of C-Nap1 to rootletin, and oligomerization of rootletin filaments.

Several other centrosome linker proteins have now been identified, including Cep68, centlein, LRRC45 and LGALS3BP (Graser et al., 2007; Fogeron et al., 2013; He et al., 2013; Fang et al., 2014). Whether these have structural or regulatory functions remains to be explored, although most are also phosphorylated by Nek2 (Man et al., 2015). Phosphorylation of linker components would keep them in a depolymerized state in mitosis, while dephosphorylation at the end of mitosis would promote linker reassembly at the start of the subsequent cell cycle. The relevant phosphatases are yet to be explored in detail. Contrary to the model whereby dephosphorylation opposes centrosome separation, overexpression of the Cdc14A phosphatase was reported to promote centrosome separation; however, the mechanism and whether this involves centrosome linker components has not been addressed (Mailand et al., 2002). It was discovered early on that Nek2 directly interacts with protein phosphatase 1 (PP1) via a KVHF motif in its C-terminal non-catalytic domain (Helps et al., 2000). However, it is unlikely that PP1 bound to Nek2 contributes to linker protein dephosphorylation at the end of mitosis as the bulk of Nek2 is degraded in mitosis as a result of ubiquitylation by the anaphase promoting complex/cyclosome (APC/C) (Hames et al., 2001; Hayes et al., 2006). In contrast, direct binding of PP1 to Nek2 may well contribute to linker protein dephosphorylation and maintenance of the centrosome linker in S and G2 when Nek2 is present and potentially active. In addition to dephosphorylating Nek2 substrates, PP1 could antagonize the auto-phosphorylation and activation of Nek2 itself (Eto et al., 2002; Mi et al., 2007). However, although Nek2 is activated by phosphorylation at T175 on its activation loop, it is not clear that this is reversed by PP1. Moreover, although Nek2 dimerises via an unusual leucine zipper motif and can auto-phosphorylate *in vitro* (Rellos et al., 2007), it remains possible that Nek2 is also phosphorylated at this site by an as yet unidentified upstream kinase.

Interestingly, the stability of the Nek2-PP1 interaction is regulated by the Plk1 kinase through a complex process that also involves the Hippo pathway kinase, Mst2 (Mardin et al., 2011). Mst2, and a scaffold protein Sav1, physically associate with Nek2, with Mst2 phosphorylating Nek2 at four sites in its non-catalytic domain. This does not obviously regulate Nek2 activity but does increase its localization to the centrosome (Mardin et al., 2010). One can speculate that this may result from increased affinity for C-Nap1 and rootletin but this has yet to be tested. Meanwhile, phosphorylation of Mst2 by Plk1 prevents association of PP1 with Nek2 leading to increased phosphorylation of Nek2 substrates, such as C-Nap1. Three sites within Mst2 are phosphorylated by Plk1. How this destabilizes the interaction of Nek2 with PP1 remains unclear, although one possibility is that Plk1 phosphorylation activates Mst2; this in turn would cause increased phosphorylation of Nek2, potentially decreasing its affinity for PP1. There is without doubt much still to be learnt about the biochemical mechanisms through which these kinases and phosphatases regulate centrosome disjunction.

Furthermore, additional protein kinases lie upstream of Nek2, Plk1, and Mst2 that contribute to centrosome disjunction. For example, Plk1 is activated by the mitotic kinase, Aurora-A, while Mst2 is under the control of the Hippo pathway kinases, Lats1/2; however, there is no evidence to date that these other kinases directly regulate Nek2. In contrast, Nek2 function is activated downstream of the EGFR tyrosine kinase. It was observed many years ago that cells exposed to EGF exhibit unscheduled separation of centrosomes (Sherline and Mascardo, 1982a,b). With the growing understanding of centrosome disjunction mechanisms, the reason for this observation was revisited with discovery that EGF regulates association of Nek2 with centrosomes via Mst2, Akt, and PI3K (Mardin et al., 2013). Further studies not only confirmed the role of Mst2 and Nek2 in EGF-mediated centrosome separation, but also demonstrated a role for the G-protein-coupled receptor kinase 2 (GRK2) in phosphorylating Mst2 in response to EGF (So et al., 2013). This occurs on two of three sites that are also phosphorylated by Plk1, hence Mst2 provides a point of integration of extracellular signals from EGFR with internal signals of mitotic progression from Plk1.

Besides inducing premature separation, overexpression of Nek2 leads to reduction in the amount of pericentriolar material (PCM) associated with interphase centrosomes (Fry et al., 1998b). This raises the question of the relationship between the centrosome linker and PCM. In many respects, these structures are considered independent. Yet this is likely to be an oversimplification, particularly considering that the timing of centrosome disjunction coincides with that of centrosome maturation when additional PCM is recruited to enhance microtubule nucleation at the onset of mitosis. A possible connection between Nek2 and centrosome maturation involves the Cdk5Rap2 protein (also called Cep215), a well-characterized PCM component that provides the scaffold for recruitment of microtubule nucleation complexes. However, Cdk5Rap2 can interact with the linker component, Cep68 (Pagan et al., 2015). Furthermore, depletion of Cdk5Rap2 not only interferes with microtubule nucleation but also leads to centrosome separation, while cells taken from Cdk5Rap2 knockout mice exhibit unpaired centrosomes indicative of loss of the centrosome linker (Graser et al., 2007; Barrera et al., 2010). Cdk5Rap2 is also a substrate of Nek2, with phosphorylation proposed to facilitate binding not only of Cdk5Rap2 but also C-Nap1 to the Wnt signaling pathway scaffold, Disheveled (DVL) (Cervenka et al., 2016). In fact DVL, and another downstream component of the Wnt pathway, β-catenin, are also Nek2 substrates and putative linker components (Mbom et al., 2014; Cervenka et al., 2016). However, based on recent studies in *Drosophila*, it's possible that these Nek2-dependent events have an entirely separate role in control of Wnt developmental signaling (Martins et al., 2017; Weber and Mlodzik, 2017). That said, as Wnt signaling is at least in part initiated at primary cilia, it is tantalizing to consider that these events may somehow link ciliary-dependent signaling with centrosome reorganization. Moreover, Nek2 has recently been shown to promote ciliary resorption through phosphorylation and activation of the microtubule depolymerizing kinesin, Kif24 (Kim et al., 2015). Hence, besides its function in centrosome disjunction, Nek2 may play roles in a number of other cell cycle-dependent processes that prepare cells for entry into mitosis.

A potentially important new piece in the jigsaw was the discovery that Nek5 might cooperate with Nek2 to regulate centrosome disjunction (Prosser et al., 2015). Localization studies suggest that a fraction of Nek5 is present centrosomes, although Nek2 is the only NEK family member detected in proteomic analyses of isolated centrosomes. Intriguingly, depletion of Nek5 disturbs centrosomes in much the same way as overexpression of Nek2, in that centrosomes exhibit both unscheduled separation and loss of pericentriolar material (PCM). However, centrosomes in cells depleted of Nek5 remained in relatively close proximity. Moreover, they had reduced levels of Nek2 and phosphorylated C-Nap1 and increased levels of rootletin suggesting that the unscheduled centrosome separation resulted from excessive recruitment, rather than disassembly, of linker proteins. Consistent with this, cells depleted of Nek5 behaved in a similar manner to cells depleted of Nek2 upon entry into mitosis in that centrosome linker proteins were not released and centrosome separation was delayed. Nek2 and Nek5 seem to not only have similar functions but cooperate in that overexpression of catalytically-inactive Nek2 blocked the separation of centrosomes that resulted from Nek5 depletion. However, we have no evidence to date that these two kinases physically interact or regulate each other's catalytic activity. Again, there is much to be learnt about Nek5, not least what acts directly upstream and downstream, before one can understand how it contributes to this process.

## NEK kinases in spindle assembly

**Figure 2:**
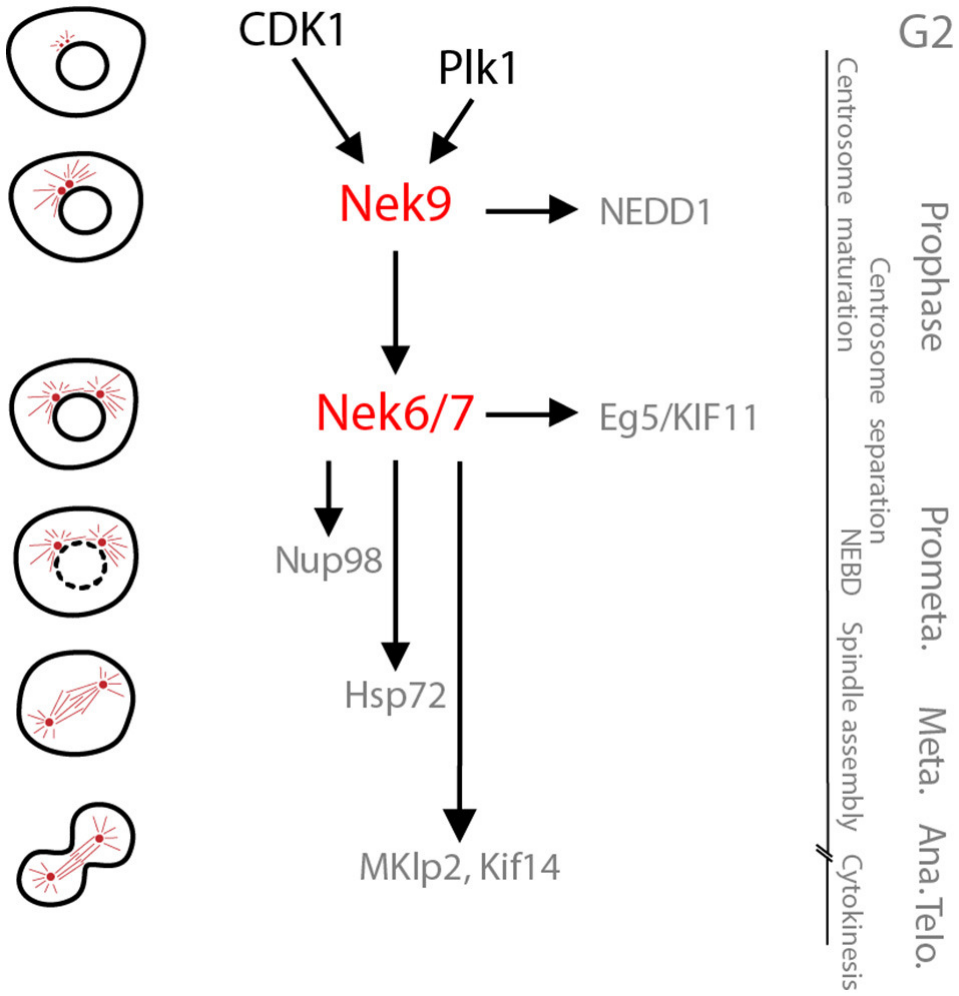
The Nek9-Nek6-Nek7 module. A schematic cartoon of the timeline of activation of the Nek9-Nek6-Nek7 signaling module and its known substrates shown with respect to the different stages of mitosis. During prophase Nek9 phosphorylates the γ-TuRC adapter NEDD1. This contributes to NEDD1 recruitment to the centrosomes and the maturation of these organelles needed for robust microtubule nucleation and spindle formation. Simultaneously, through activation of Nek6 and Nek7, Nek9 controls recruitment of the kinesin Eg5 to the centrosomes that leads to their separation. Nek6 and Nek7 are also involved in nuclear envelope breakdown (NEBD) through phosphorylation of the Nup98 nucleoporin, and subsequently in spindle organization through phosphorylation of Hsp72. Finally, Nek6 and Nek7 are involved in the control of cytokinesis via regulation of the kinesins, Mklp2 and Kif14. Note that some of the substrates attributed to Nek6 and Nek7 may be specific for one of these two kinases (e.g., Hsp72 and Mklp2 for Nek6, and Kif14 for Nek7). *Meta, Ana, and Telo*, metaphase, anaphase and telophase.

As cells progress into mitosis and Nek2 activity is shut down through proteasome-mediated degradation (Hames et al., 2001; Hayes et al., 2006), a signaling module composed of Nek9, Nek6, and Nek7 becomes active (Figure 2). These three NIMA-related kinases also perform essential functions in spindle assembly, and are switched on through a series of molecular events that are set in motion upon activation of CDK1 (Bertran et al., 2011). CDK1 phosphorylates Nek9 at Ser-869 to create a binding site for the polo-box domain of Plk1. Plk1 can then phosphorylate Thr-210 in the activation loop within the catalytic domain of Nek9. This results in activation of Nek9 and in turn stimulates autophosphorylation in its C-terminal non-catalytic region that enables it to bind directly to Nek6 and Nek7. This leads to activation of Nek6 and Nek7 through both allosteric and non-allosteric mechanisms. Thus, Nek2, Nek9, Nek6, and Nek7 activation depends on Plk1 defining the specific time window at the onset of mitosis when these kinases are turned on. Whether this relationship between NEKs and Polo-family kinases is conserved in other organisms remains to be established.

Nek6 and Nek7 were identified in 2000 as two NIMA-related kinases with highly similar (85% identical) catalytic domains (Kandli et al., 2000). Initial experiments suggested that Nek6 and Nek7 might be activators of the interphase p70 S6 kinase as they are both able to efficiently phosphorylate its hydrophobic regulatory site *in vitro* (Belham et al., 2001). However, subsequent work ruled this out as a physiological role for Nek6 and Nek7 (Lizcano et al., 2002). In fact, they were found to interact with Nek9, which acts upstream as a kinase able to phosphorylate and activate Nek6 and Nek7, and evidence emerged indicating a role for these three kinases in mitosis (Roig et al., 2002; Belham et al., 2003; Yin et al., 2003).

Nek6 and Nek7 are the shortest members of the family, consisting of 313 and 302 residues, respectively, in both humans and mice. In contrast to the other NEKs, their sequence lacks obvious regulatory domains and is almost entirely composed of a catalytic kinase domain. The only region of significant difference is the short (30–40 residue) extensions that are N-terminal to the kinase domains. Interestingly, there is now evidence that these N-terminal extensions provide specificity toward substrates and other binding partners in different cellular contexts (Vaz Meirelles et al., 2010). Indeed, as explained below, the majority (but not all) of Nek6 and Nek7 substrates identified to date are specific for one or other kinase. Curiously though, most of the functions described for Nek6 and Nek7, at least in terms of mitotic progression in cancer cell lines, are very similar. However, consistent with the concept of targeting different substrates, the two kinases are not redundant as loss of either protein in model transformed cell lines leads to mitotic arrest and cell death.

Functional specificity may also come from different expression patterns in specific cell or tissue types. High-throughput transcriptomic analyses show that human and mouse Nek6 and Nek7 mRNAs are present in most tissues, albeit with minor variations in levels (https://www.ncbi.nlm.nih.gov/gene). However, more targeted studies indicate that the two kinases may be expressed differentially during embryogenesis and in different regions of the adult nervous system (Feige and Motro, 2002). Consistent with this, elimination of either kinase in transgenic animals has radically different effects. Nek6 knockout animals are born at Mendelian ratios and do not show an obvious phenotype, at least when unchallenged (http://www.informatics.jax.org/marker/MGI:1891638), although they do exhibit increased cardiac hypertrophy after transthoracic aorta constriction (Bian et al., 2014). In contrast, knockout of Nek7 results in late embryonic or perinatal lethality and severe growth retardation (Salem et al., 2010). Although the cause of this is unknown, it suggests that Nek7 has crucial functions during development that cannot be replaced by Nek6.

Nek6 and Nek7 have a number of different functions during mitosis related to the control of centrosome positioning, spindle assembly and cytokinesis (Figure 2). Both Nek6 and Nek7 exhibit weak localization to spindle poles, while Nek6 has also been detected on spindle microtubules (Yissachar et al., 2006; Kim et al., 2007; O'Regan and Fry, 2009). Meanwhile, activated Nek9 as detected with a Nek9-pT210 antibody is present on centrosomes during mitosis (Roig et al., 2002, 2005; Belham et al., 2003). The expression of Nek6, and possibly Nek7, appears to increase as cells approach mitosis; however, more obvious is a gel shift on Western blots of both kinases in mitosis that is presumably indicative of activation (Belham et al., 2003; O'Regan and Fry, 2009). Interference with Nek6 or Nek7 results in an increase of mitotic cells, abnormal chromosome segregation, multinucleation and cell death (Yin et al., 2003; Yissachar et al., 2006; O'Regan and Fry, 2009). Early experiments expressing loss-of-function mutants suggested that this reflects multiple, independent functions as mutants devoid of kinase activity blocked cells at metaphase through activation of the spindle assembly checkpoint, while hypomorphic mutants allowed progression through metaphase but prevented completion of cytokinesis (O'Regan and Fry, 2009). More recent studies based on RNAi-mediated depletion have revealed a number of different substrates of these kinases that may well explain these observations.

First, downstream of Nek9, Nek6, and Nek7 control the separation of centrosomes in prophase by regulating the spindle pole localization of the kinesin Eg5 (Rapley et al., 2008; Bertran et al., 2011). Nek6 and Nek7 phosphorylate Eg5 at a specific site (Ser-1033 in humans) that, together with CDK1 phosphorylation at a site necessary for microtubule interaction (Thr-926), allows Eg5 to accumulate around centrosomes and stimulate their separation before nuclear envelope breakdown^1^. This favors timely and accurate chromosome segregation (Silkworth et al., 2012). Second, both Nek6 and Nek7 contribute to nuclear envelope breakdown though phosphorylation of the nuclear pore protein, Nup98 (Laurell et al., 2011). In prometaphase and metaphase, Nek6 and Nek7 are then required for robust spindle assembly (O'Regan and Fry, 2009). Although the role of Nek7 in this process remains to be determined, one mechanism by which Nek6 promotes spindle assembly is through phosphorylation of the heat shock protein, Hsp72 (O'Regan et al., 2015). Hsp72 phosphorylation by Nek6 is necessary for recruitment of ch-TOG and TACC3 to kinetochore associated microtubules, or K-fibres. However, whether phosphorylated Hsp72 stabilizes interaction of ch-TOG and TACC3 and their subsequent binding to microtubule plus-ends, or promotes their association with clathrin and formation of inter-microtubule bridges is unclear (Hood et al., 2013; Gutiérrez-Caballero et al., 2015). Nek6 and Hsp72 are also required for centrosome clustering in cancer cells with amplified centrosomes, presumably through similar mechanisms to those that promote K-fiber stabilization (Sampson et al., 2017). Finally, several reports implicate Nek6 and Nek7 in the control of cytokinesis (Rapley et al., 2008; O'Regan and Fry, 2009; Salem et al., 2010; Cullati et al., 2017). Assuming that Ser-1033 on Eg5 is exclusively phosphorylated by Nek6, then a phosphospecific antibody raised against this site suggests that Nek6 activity peaks in late mitosis. Moreover, Nek6 and Nek7 phosphorylate distinct kinesins, namely Mklp2 and Kif14, to directly regulate cytokinesis. Specifically, phosphorylation by Nek7 stimulates Kif14 activity and recruitment of the Rho-interacting kinase, citron, to the spindle midzone in anaphase, while phosphorylation by Nek6 controls Mklp2 localization and its microtubule bundling activity at the central spindle in telophase.

Although the total cellular levels of Nek6 and Nek7 kinase activity peak in mitosis, there is growing evidence of additional roles for these kinases outside of mitosis. For example, Nek7 may be important for the cell cycle-dependent regulation of primary cilia, as *Nek7*^−/−^ MEFs exhibit abnormal cilia numbers (Salem et al., 2010). Moreover, careful measurement of microtubule dynamics supports a role for Nek7 in regulating microtubules in interphase (Cohen et al., 2013). Indeed, both Nek6 and Nek7 are capable of directly phosphorylating microtubules *in vitro* (O'Regan and Fry, 2009). Furthermore, Nek6 and Nek7 have been implicated in regulation of centrosome duplication and maturation, senescence and the DNA damage response, all of which primarily take place in interphase (Lee et al., 2008; Jee et al., 2010; Kim et al., 2011; Gupta et al., 2017; Tan et al., 2017). Unexpectedly, Nek7 was found to be necessary for activation of the NLRP3 inflammasome, a multiprotein complex that activates inflammatory caspases in macrophages (He et al., 2016; Schmid-Burgk et al., 2016; Shi et al., 2016). This role is independent of its kinase activity and may be important to ensure that inflammasome activation is mutually exclusive with mitotic progression.

Nek9 was originally identified through coimmunoprecipitation with Nek6 from cultured cell lines (Roig et al., 2002). In parallel, it was purified during a search for protein kinases induced by IL-1, although it is not activated by the interleukin (Holland et al., 2002; in this paper, the kinase was misnamed as Nek8). Nek9 is one of the longer NEKs, being ~1,000 residues in length (979 in humans, 984 in mice). The non-catalytic C-terminal region of Nek9 begins with an RCC1 domain that is similar in sequence to the Ran exchange factor, RCC1, and has led to Nek9 sometimes being referred to as Nercc1. This is followed by a C-terminal tail that contains a number of S/TP and PXXP motifs, a region that binds to Nek6 and Nek7 as well as the multifunctional dynein light chain LC8, and a coiled-coil that acts as an oligomerization motif. Nek9 is able to undergo autophosphorylation and activation *in vitro* in a manner that is dependent on the coiled-coil motif, while the RCC1 domain acts as an autoinhibitory domain (Roig et al., 2002). As indicated above, Nek9 is inactive in interphase and activated during mitosis by a two-step mechanism involving CDK1 and Plk1 (Roig et al., 2005; Bertran et al., 2011). Active Nek9 undergoes autophosphorylation at a number of sites, one of which interferes with binding to LC8 (Regué et al., 2011; Gallego et al., 2013). LC8 constitutively binds to inactive, unphosphorylated Nek9 impeding its interaction with Nek6 and Nek7. Hence, it is only upon activation and autophosphorylation that Nek9 can bind and activate Nek6 and Nek7. Whether LC8 completely prevents interaction of Nek9 with Nek6 and Nek7 in interphase, and the mechanism through which the RCC1 domain acts in an auto-inhibitory manner are important and unanswered questions.

Like Nek6 and Nek7, Nek9 is necessary for mitotic progression, spindle formation and chromosome segregation (Roig et al., 2002, 2005; Kaneta and Ullrich, 2013). Being able to activate the closely-related Nek6 and Nek7 kinases, means that conceptually these functions of Nek9 could operate entirely through activation of these two downstream kinases. In reality though, additional substrates regulated independently of Nek6 and Nek7 have been identified (Figure 2). In this regard, it is worth remembering that Nek6 and Nek7 may have Nek9-independent roles both in interphase and mitosis that rely on alternative activation mechanisms. Specific Nek9 functions have been identified using antibody microinjection and RNAi in cultured cells, as well as by immunodepletion of the *Xenopus* Nek9 ortholog from egg extracts. These have revealed the importance of Nek9 in regulation of centrosome maturation and separation, two steps that occur after Nek2-induced centrosome disjunction. The role of Nek9 in centrosome maturation is independent of Nek6 and Nek7 and depends on the direct phosphorylation of NEDD1, an adaptor of the microtubule nucleating γ-tubulin ring complex (γ-TuRC) (Sdelci et al., 2012). Centrosome maturation results from accumulation of γ-TuRC and other components of the pericentriolar material (PCM) and provides the centrosome with the additional microtubule nucleating capacity needed for spindle organization in mitosis (Fry et al., 2017). Nek9 phosphorylates NEDD1 at a site that drives its recruitment to the PCM although the mechanism remains unclear. This makes Nek9 a key regulator of centrosome maturation together with Plk1 (Haren et al., 2009). Importantly, centrosome maturation occurs simultaneously to the regulation of centrosome separation, which is also regulated by Nek9 through the Nek6/Nek7-dependent phosphorylation of Eg5, as described above (Rapley et al., 2008; Bertran et al., 2011).

Centrosome maturation and separation are important processes for efficient mitotic progression, and may explain the observed effects on mitosis of interfering with Nek9 activity. However, it is likely that other functions that contribute to the observed effects of interfering with Nek9 in organisms await discovery. Nek9 is ubiquitously expressed (Roig et al., 2002; https://www.ncbi.nlm.nih.gov/gene) and its functions are likely to be crucial during development based on the recent description of a Nek9 mutation in humans (c.1489C>T; p.Arg497^*^) that is associated with a high frequency of abortions (Casey et al., 2016). At the cellular level, this may be explained by a significant reduction in cell proliferation, as similar truncation mutants of Nek9 that retain the kinase domain but lack the Plk1 binding region and coiled-coil are inactive (Roig et al., 2002). Nevertheless, the possibilities that the mutant could be activated or have kinase-independent roles cannot be ruled out.

## Biochemical regulation of mitotic NEK kinases

Structural and biochemical studies have yielded insights into the mechanisms by which NEK kinases are regulated, informed by comparisons with other families of protein kinases. The catalytic domains of protein kinases consist of an N-lobe and a C-lobe joined by a flexible hinge. These three structural elements form a deep pocket into which ATP binds. In common with other protein kinases, the structural and catalytic cores of NEKs consist of conserved sequence motifs: the catalytic motifs [His-Arg-Asp (HRD) and Asp-Phe-Gly (DFG)]; the Lys-Glu salt bridge within the N-lobe; the regulatory spine of hydrophobic core residues (R-spine); and the activation loop in which the site(s) of activating phosphorylation are located (Bayliss et al., 2012). Structures of kinases in their active states show these motifs positioned ready for catalysis, while they are displaced in inactive kinase states. Crystal structures of NEK catalytic domains solved to date all reveal stable structural features that would be expected to block activity, indicating auto-inhibited states that require energetic input to allow activation of the kinase. The process of kinase activation may involve phosphorylation and/or protein-protein interactions. Indeed, activation loop phosphorylation is critical for the activity of NEK kinases, as evidenced by the reduction of activity observed when activation loop Ser/Thr residues are mutated to Ala (Belham et al., 2003; Roig et al., 2005; O'Regan and Fry, 2009; Zalli et al., 2012). However, all crystal structures of NEKs have used protein that lacks this post-translational modification, and so we have not yet observed a NEK in its active conformation.

The first structure of a NEK kinase was that of human Nek2, obtained in the presence of the ATP-competitive inhibitor SU11652 (Rellos et al., 2007). Subsequent structures of Nek2 were obtained in complex with ADP, a non-hydrolyzable ATP analog or a number of other inhibitors (Richards et al., 2009; Westwood et al., 2009; Solanki et al., 2011). These structures showed that, without phosphorylation on the activation loop, this region of the kinase might adopt several different conformations, most of which do not resemble that expected for an active kinase. Much of the activation loop is disordered, while the N-terminal section of this loop forms an α-helix in a subset of structures, proposed to be an auto-inhibitory feature that must be unwound for kinase activity. Furthermore, the other key features are consistent with an inactive kinase: the Lys-Glu salt-bridge is broken, the R-spine is not formed, and the DFG and HRD motifs are out of the positions required for catalytic reaction. The closest to an active structure of Nek2 is found in complex with a series of ATP-competitive inhibitors based on a purine scaffold (Coxon et al., 2017). These show the HRD and DFG motifs in approximately the right positions for catalysis, and the R-spine is almost formed, but in other respects the conformation remains that of an inactive kinase. Phosphorylation of Nek2 most likely drives the formation of an active conformation through interactions between the phosphate group attached to Thr175 and two basic residues on the activation loop and HRD motif (Bayliss et al., 2012). However, the physiological mechanism of Nek2 activation might involve more than one phosphorylation event in the activation loop because individual phospho-mimic mutations of T170E, S171D, T175E all increase activity (Rellos et al., 2007).

The catalytic domain of human Nek1 kinase has also been crystallized and the structure determined in apo-form and in the presence of an ATP-competitive inhibitor. The protein used had a T162A mutation, the principal site of activating phosphorylation, and was therefore in an inactive state. Unlike Nek2, the activation loop of inactive Nek1 was fully ordered, albeit in a conformation that is incompatible with substrate binding (Melo-Hanchuk et al., 2017). The region of the activation loop in the vicinity of residue 162 formed an α-helix. Like Nek2, this helix might be an auto-inhibitory feature, but the structural basis of Nek1 activation remains to be discovered.

**Figure 3:**
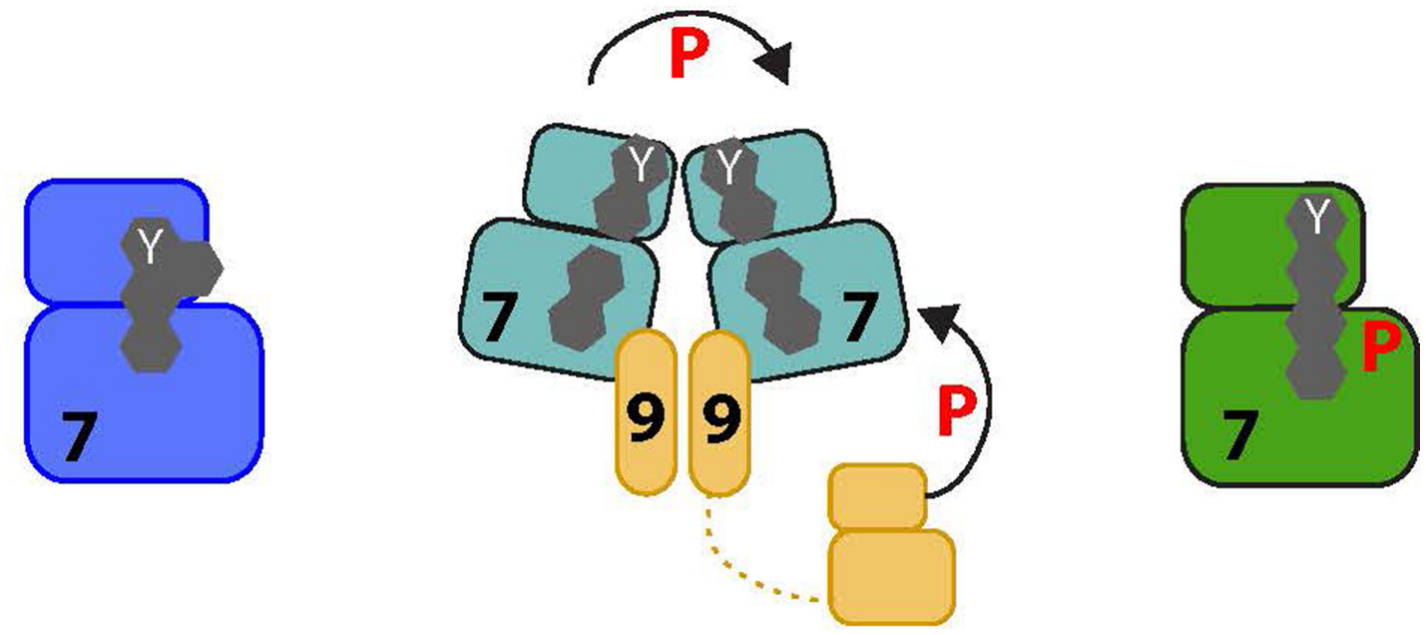
Proposed activation pathway of NEK7. The four amino acids that form the R-spine of Nek7 are shown as gray hexagons. On the left, inactive Nek7 is shown with a condensed R-spine in which the side chain of Tyr97 (Y) is located in the interior of the protein. This is a stable conformation that prevents formation of a productive kinase active site. In the center, dimeric Nek9 is shown interacting with two molecules of Nek7, bringing them together in a back-to-back conformation that destabilizes the inactive conformation, promoting Nek7 autophosphorylation. Nek9 can also activate Nek7 through direct phosphorylation of the activation loop. On the right, Nek7 is shown in a catalytically active state with phosphorylation on Ser195 and, we predict, alignment of the R-spine residues.

Nek6 and Nek7 activity depends on the phosphorylation of their activation loops at Ser206 and Ser195 respectively (Belham et al., 2003; O'Regan and Fry, 2009). The crystal structure of unphosphorylated human Nek7 revealed an unexpected autoinhibitory mechanism in which the side chain of the top R-spine residue, Tyr97, points into the active site stabilizing the inactive state of the kinase (Richards et al., 2009; Figure 3). The kinase is activated by binding of the C-terminal, non-catalytic domain of Nek9 that dimerises through a coiled-coil domain and could thereby bring together two molecules of Nek7 to promote autophosphorylation. The molecular mechanism was resolved in the structure of Nek7 in complex with a short peptide from Nek9 (Haq et al., 2015). In this structure, Nek7 forms a back-to-back homodimer with an interface centered on the αC-β4 loops in the vicinity of residue 97 (note that Tyr97 was mutated to phenylalanine to generate crystals). Back-to-back dimerization is coupled to conformational changes that activate the kinase through rearrangements of the R-spine, accelerating the process of autophosphorylation, which is slow in the absence of Nek9 (Dodson et al., 2013). Nek7 phosphorylated on Ser195 is already active, and addition of Nek9 does not stimulate activity further (Rogerson et al., 2015). The Nek9 binding site of Nek7 is conserved on Nek6, which could be regulated through a similar mechanism. Interestingly, the back-to-back dimerization surface of Nek7 is poorly conserved in Nek6, and so activation of these kinases through heterodimerization via Nek9 is unlikely providing an explanation for how these highly related kinases could be independently activated (or inhibited). Ultimately though, as Nek9 is able to directly phosphorylate Nek6 and Nek7 on their activation loops, the relative contribution of direct phosphorylation by Nek9 versus Nek9-stimulated autophosphorylation in cells is not known.

The preference of Nek2 for substrates with a Phe, or another hydrophobic amino acid in the P-3 position, can be rationalized based on crystal structures (Alexander et al., 2011). This residue is predicted to bind into a pocket formed by a pair of alanine residues (95 and 145), a combination that is rare in other kinases. Interestingly, the equivalent positions in Nek6, Nek7 and Nek8 are also small amino acids, and Nek6 at least also has a preference for substrates with a hydrophobic residue at P-3 (Lizcano et al., 2002). In contrast, we predict that other human Nek family kinases may not share the same substrate preference because the pocket that recognizes the P-3 residue is blocked by bulkier amino acids.

## Future perspectives

There remain many unanswered questions regarding the biological functions of NEKs, as well as their contributions to disease mechanisms. In terms of centrosome disjunction, we do not fully understand how NEKs regulate the organization and length of the centrosome linker, or its connection to centrioles. Besides C-Nap1 and rootletlin, there are a growing number of proteins that have been proposed to be part of the linker but we know little about their relative importance or regulation. Similarly, while we have made good progress in identifying substrates of Nek2, we have only just uncovered a role for Nek5 in centrosome disjunction. We therefore need to identify both downstream substrates and upstream regulators of Nek5 to understand how it might cooperate with Nek2. In addition, we need to explore how centrosome disjunction is coordinated with other changes in centrosome organization during cell cycle progression, including ciliary resorption and centrosome maturation. It will be equally important to investigate how the centrosome linker is reassembled in late mitosis and how this is coordinated with disengagement of the duplicated centriole pair. In this regard, we hypothesize that there may be competition between C-Nap1 and the centriole duplication factor, SAS-6, for binding to Cep135, and that timely degradation of SAS-6 at the end of mitosis may be necessary for recruitment of C-Nap1 and establishment of a new linker (Strnad et al., 2007; Lin et al., 2013). Furthermore, a number of extracellular-mediated signaling pathways, including the Hippo, EGFR, PI3K, and Wnt pathways, impact on Nek2 function, and it will be intriguing to explore whether this relates to control of centrosome disjunction or rather flags alternative roles for this kinase in proliferation.

Regarding Nek9, Nek6, and Nek7, one of the major challenges will be to understand their relative importance in different cell types and tissues, and whether Nek6 and Nek7 have redundant functions in specific physiological contexts. Indeed, the role of these kinases in normal development needs to be clarified, particularly in light of data showing that mutations in human Nek9 lead to either abortions (Casey et al., 2016) or malformations (Shaheen et al., 2016). It will also be important to determine whether Nek6 and Nek7 activation absolutely depends on Nek9, or whether there are alternative mechanisms for activation of the two smallest NEKs. Whether some of Nek6, Nek7, or Nek9 functions are independent of their catalytic activity should also be considered. This will clarify whether the kinase-independent role of Nek7 in the inflammasome is an exceptional case driven by a need to prevent mitosis by sequestration of the kinase during the inflammatory response (Shi et al., 2016). We should determine whether Nek9 has other Nek6 and Nek7 independent functions in addition to phosphorylation of NEDD1. Indeed, an interesting question is whether Nek9 has a role in the nucleus as a fraction of Nek9 has been reported to be associated with the chromatin modulator, FACT (Tan and Lee, 2004). An additional area of study is how Nek9, Nek6, and Nek7 are turned off at the end of mitosis, whether this relies on phosphatases or the degradation of active kinases, and possible pathological consequences of a failure to inactivate the kinases.

Finally, our understanding of the structural mechanisms within NEK kinase pathways is far from complete. For instance, there is no experimental structural model of a NEK kinase in its active state, principally because protein samples are heterogeneously phosphorylated and unstable when expressed in recombinant form. This issue may be resolved by using genetically encoded phosphorylation to generate purified proteins with phosphorylation on specified serines or threonines. This approach could also be applied to the substrates of NEKs, such as Hsp72, to enable studies on the structural and functional consequences of phosphorylation. To date, structural studies on NEKs have been restricted to individual domains and the complex of Nek7 with a short peptide derived from Nek9, whereas most NEKs are multi-domain proteins. A major aim of future work is to resolve the structures of full-length NEKs that include their *in cis* regulatory motifs, such as the Nek2 leucine zipper (Croasdale et al., 2011) or the Nek9 RCC1 domain (Roig et al., 2002), and larger-scale regulatory complexes. This could be done using cryo-electron microscopy, but first some effort is required to produce these challenging protein samples with sufficient yield, purity and stability. Finally, there are very few potent inhibitors of NEK kinases, and the selectivity of these compounds has not been tested across the entire family. Our understanding of NEK biology would be transformed if a toolkit of potent and selective chemical inhibitors were available. Fortunately, the lack of chemical probes for many protein kinases is now widely recognized and efforts are underway to develop these essential tools.

## Author contributions

AF, RB, and JR contributed to the planning and writing of this review article.

### Conflict of interest statement

The authors declare that the research was conducted in the absence of any commercial or financial relationships that could be construed as a potential conflict of interest.

## Footnotes

^1^Nek9-dependent phosphorylation of Eg5 has recently been shown to regulate binding of this kinesin to the multifunctional microtubule-associated protein, TPX2. In addition, Nek9 regulates the centrosomal localization of Eg5 in prophase by phosphorylating and maintaining a pool of TPX2 at centrosomes before NEBD (Eibes et al., in press).
